# The Nutriepigenome

**DOI:** 10.3390/genes14111997

**Published:** 2023-10-25

**Authors:** Mario G. Mirisola

**Affiliations:** STeBiCeF Department, Università di Palermo, Building 16, Viale delle Scienze, 90128 Palermo, Italy; mario.mirisola@unipa.it

**Keywords:** epigenetic diet, nutrients affecting epigenome, phytochemicals, functional diet

## Abstract

Unlike genetic changes, epigenetics modulates gene expression without stable modification of the genome. Even though all cells, including sperm and egg, have an epigenome pattern, most of these modifications occur during lifetime and interestingly, some of them, are reversible. Lifestyle and especially nutrients as well as diet regimens are presently gaining importance due to their ability to affect the epigenome. On the other hand, since the epigenome profoundly affects gene expression profile it can be speculated that the epigenome could modulate individual response to nutrients. Recent years have thus seen growing interest on nutrients, macronutrients ratio and diet regimens capable to affect the epigenetic pattern. In fact, while genetic alterations are mostly detrimental at the individual level, reshaping the epigenome may be a feasible strategy to positively counteract the detrimental effect of aging. Here, I review nutrient consumption and diet regimens as a possible strategy to counteract aging-driven epigenome derangement.

## 1. Epigenome Machinery

The DNA methyltransferases (DNMTs) are a gene family whose protein products methylate the C5 position of genomic cytosines. DNMTs preferentially target the dinucleotide CpG and, as a result, 70% of all CpG dinucleotides are found methylated in somatic cell lines. CpG islands, often found in regulatory regions of protein coding genes [1], usually inhibit transcription when methylated.

Non CpG DNA methylation (CC, CT and CA) has been described in neurons, skeletal muscle, T-cell and oocytes as well as in embryonic stem cells and pluripotent stem cells where accounts 15% of the total methylated cytosines but seems to be less abundant in faster dividing tissues [1,2,3,4]. 

DNMTs activation and inhibition are the major regulators of methylation and demethylation both of CpG and non CpG sites. In addition, methylated cytosines can be oxidized by a group of enzymes, the TET enzymes, giving rise to several intermediate products. It is interesting to note these intermediates, present in very low concentration, are promptly affected by environmental signals [5]. We can therefore speculate that, in addition to methylation and demethylation status, these intermediates constitute additional epigenetic signals particularly sensitive to environmental stimuli. Methylation status can thus be considered a dynamic equilibrium between methylation, demethylation and in between with derangement of this fine-tuned mechanism associated to pathological conditions. For example, global demethylation and the resulting genomic instability are considered a hallmark of aging and cancer [6], while increased methylation level is observed in the presence of congenital defects such as down syndrome and gestational diabetes [7]. 

A second level of epigenetic regulation is posttranslational modification of histone proteins. These small proteins, chemically basic, are abundant in the nucleus being an essential part of nucleosomes, the structural unit of chromatin. Each nucleosome encompasses 8 proteins, the histone octamer, wrapped by a short segment (147 bp) of DNA. A DNA linker of variable length connects neighboring nucleosomes and binds another histone, called H1. Each histone octamer is constituted by 2 H2A-H2B dimers and one 2X (H3-H4) tetramer. N terminal tails of each octameric histone are exposed and subjected to several posttranslational modifications including acetylation, methylation, sumoylation, phosphorylation, ubiquitination [8]. It is interesting to note that while DNA methylation is relatively stable, histone posttranslational modifications change rapidly in response to internal as well as external stimuli [9]. Acetylation of histone proteins is generally associated with reduced affinity for DNA and thus increased accessibility for transcriptional machinery. It is catalyzed by the protein products of the histone acetyltransferases gene family (HAT) and counterbalanced by the activity of the histone deacetylases proteins (HDAC). Methylation of histones also occurs and can have both activating and repressing effects, depending on the amino acid involved [9]. Several substances affect histone epigenetics: tobacco smoke, cadmium and lead are examples of such substances [10].

Beside the enzymatic activity of HAT and HDAC gene products, methylation as well as demethylation of both DNA and histone proteins need the availability of specific molecules capable to donate or accept methyl groups respectively. The concentration of these donor and acceptor molecules may vary and can force the process toward methylation or demethylation. An example of such molecules is S-adenosylmethionine (SAM). This is the methyl group donor in methylation of both DNA and histones. Although the ribose 5-phosphate, needed for this process, is recycled, its continuous usage for nucleotide synthesis, especially in fast growing tissues links its availability to the phosphate pentose pathway, a process driven and thereby dependent on glucose availability [11]. In addition, the SAM synthesis requires the presence of methionine [12], and it is thus connected to folate as well as homocysteine metabolism. In fact, even though methionine is recycled through 1-carbon metabolism, the drainage by protein synthesis makes it an essential nutrient. Intracellular glucose and methionine thus play a role on the efficiency of SAM synthesis and are in turn affected by glycolysis, amino acid metabolism, choline [13] and folate availability [14]. These interconnections constitute a link between cellular metabolism and epigenome. In fact, methionine restriction, which can be easily obtained excluding animal protein sources from the diet, results in decreased global methylation in cancer cells [15] and mice fed with increasing amount of methionine have a correspondent increase in SAM availability [16]. However, because of the cyclical nature of 1-carbon metabolism, excess of methionine may inhibit the methylation of homocysteine maintaining the equilibrium between s-adenosylhomocysteine and s-adenosylmethionine [17,18,19]. Animal studies measuring methionine, s-adenosyl methionine and s-adenosyl homocysteine levels after administration of different amounts of methionine have given mixed results often with tissue-specific differences [16,17,18]. The observed discrepancies on the effects of methionine supplementation or deprivation on SAM availability suggest the existence of a complex relation between methionine administration and SAM availability likely dependent on tissue, age and duration of exposure [20]. On the contrary demethylases and TET enzymes require alpha ketoglutarate (α-KG) as cofactor. The latter is an intermediate of the Krebs cycle, but it is also synthesized by glutamine transamination. It has recently been demonstrated, in yeast model, that histone methylation affects the nuclear ketoglutarate availability suggesting the existence of a feedback mechanism balancing the concentration of this methyl group acceptor [21]. The concentration of α−KG it is also dependent on metabolism of glutamine, branched chain amino acids and serine [11]. Manipulation of the concentration of these amino acids can affect the availability of α-KG altering the efficiency of the demethylation process [11]. In addition, since the Jumanji histone demethylases need Fe2+ as a cofactor and asparagine deficiencies reduce the Fe2+ availability, asparagine depletion can also indirectly affect the efficiency of the demethylation process [22]. However, since normal cells are capable to synthetize asparagine, the latter effect is normally masked and can only be seen in cells which have lost this synthetic capability like acute leukemia cells [22]. Since the availability of glucose, methionine, amino acids as well as the efficiency of Krebs cycle may rely on external factors such as nutrient supply, the epigenome may be dependent on cellular metabolism, external stimuli as well affected by the availability (ratio?) of the methyl donor and acceptor molecules.

Another player of the epigenetic landscape are non-coding RNAs (ncRNA). These are a heterogeneous group of molecules, with size-dependent classification, constituting the RNA molecules with the highest complexity in mammals. They have several roles in multiple steps of protein synthesis (e.g., tRNA and rRNA), gene expression (miRNA) [23], chromatin assembly, silencing of repetitive sequences and centromere function (lncRNA) [24]. 

Many environmental factors affect epigenetic mechanism through ncRNA. A clear example is constituted by heavy metals lead and cadmium which have been related to some neurological disorders as Alzheimer’s disease, amyotrophic lateral sclerosis and Parkinson’s disease with a mechanism involving ncRNA impairment [24] (Table 1).

## 2. Substances Affecting the Epigenome

As stated above, global DNA demethylation has been associated with chromosomal instability and considered a hallmark of aging and of several types of cancers. It is therefore of great interest the identification of natural substances capable to delay or even revert the epigenome derangement of aging and cancer. 

Global modulation of the epigenome has been associated with the presence- absence of some substances. For example, insufficient availability of zinc resulted in decreased SAM-dependent methylation of rat liver leading to global DNA hypomethylation [28]. Similar results were also obtained with selenium deficiency both on Caco2 cells and in mice experiments [29]. Curiously, both deficient and excessive amount of arsenic are associated with DNA hypomethylation suggesting a complex relationship of this substance with epigenome regulation [30]. On the contrary, increased amount of retinoic acid, a vitamin A active metabolite, led to genome hypomethylation and is associated with greater incidence of tumor [28]. Since methylation occurrence is linked to SAM availability, which is the only methyl donor in DNA, histone and RNA methylation processes, its concentration is obviously crucial for any methylation process. As stated in the preceding paragraph, many factors may affect SAM concentration potentially contributing to epigenome regulation. The 1-Carbon metabolism as well as nutrients like vitamin B6, B12, riboflavin, betaine, folate and choline, amino acids methionine, cysteine, serine and glycine take part in SAM biosynthesis and are believed capable to affect its concentration. This hypothesis is confirmed by the observation that insufficient availability in some of them associates with DNA hypomethylation. For example, folate deficiency and replenishment are associated with decreased and increased DNA methylation respectively [31]. Interestingly, a study made in older women revealed that folate replenishment didn’t fully restore methylation status suggesting aging may limit the reversibility of this process [32]. 

The methylenetetrahydrofolate reductase (MTHFR) gene, whose mutations are implied in newborn abnormalities can also affect SAM concentration. Two mutations of this gene are known at position 677 (C677T) and 1298 (A1298C). Homozygosity or compound heterozygosity with these mutations are associated to increased need of folate supplementation to maintain normal concentration in blood. One of these mutations, C677T, increases the risk of colorectal cancer but only if folate deficiency is not counterbalanced by folate supplementation [33]. Interestingly, this condition associates with genomic hypomethylation [33].

**Figure 1:**
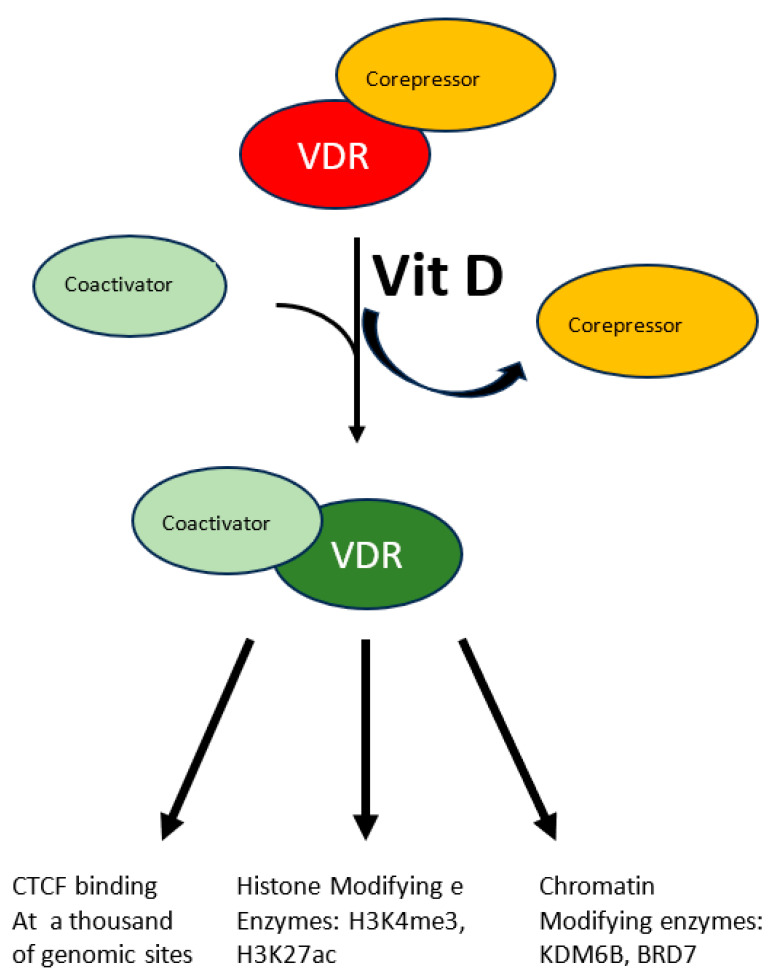
The multiple effects of vitamin D. The interaction between vitamin D and its receptor (VDR) induces the latter to switch from the corepressor to the coactivator-bound form. Activated VDR acts at multiple levels to modify chromatin status: CTCF activation, histone post-translational modification and chromatin’s 3D structure.

A more specific modulation of the epigenome has been demonstrated in the case of vitamin D. Beside its fully demonstrated role in bone mineralization and immune modulation, emerging roles as modulator of oxidative stress are appearing for this molecule which is involved in several diseases. This vitamin, mainly introduced eating fat fish, can also be produced by our skin through the conversion of 7-dehydrocholesterol to vitamin D3 in response to UVB solar radiation. The resulting hydrophobic substance, whose blood solubility is assured by the carrier Vitamin D binding protein, is transported from keratinocytes where it is produced or from enterocytes where it is absorbed to the liver where it undergoes the first hydroxylation reaction, it is then transported to proximal tubules of kidneys where the active hormone is finally produced by a second hydroxylation reaction. Cells of the innate immune system can also do the last modification and locally use the active hormone mostly by paracrine or autocrine signaling. The active hormone targets all tissue expressing the nuclear vitamin D receptor (VDR). In response to the ligand-receptor binary complex, VDR reshapes changing the affinity balance from corepressor to coactivator proteins (see Figure 1) [34]. Interaction between activated VDR and chromatin modifying enzymes such as KDM6B and BRD7 has been demonstrated as well as H3K4me3 and H3K27ac mark the chromatin response to vitamin D [34]. Even though the molecular characterization of the vitamin D-triggered epigenetic modifications is at its beginning, in vitro studies with human monocytes have demonstrated increased accessibility to 4500 chromatin loci, most of which in promoter or enhancer regions [34], in response to vitamin D treatment, suggesting an important role of this vitamin on a wide range of epigenetic targets. Vitamin D is also capable to modulate CTCF binding at about 1300 genomic sites. This zinc finger protein, highly conserved, regulates not only transcription activation or repression depending on the target site, but also chromatin 3D structure. In summary modulation of the epigenome is accomplished by vitamin D through modulation of transcription factor binding as well as chromatin accessibility, histone marker and 3D chromatin organization.

Interestingly vitamin D response index vary throughout the population. Like for the response variability to other molecules it is thought that genetic polymorphisms may have a role. The enzymatic activity of 7-dehydrocholesterol reductase which converts 7-dehydrocholesterol into cholesterol is an example. In fact, depending on its enzymatic activity, it subtracts more or less substrate for the UVB-mediated vitamin D3 synthesis. Consequently, genetic mutation lowering its enzymatic activity increases the endogenous synthesis of vitamin D3.

Vitamins C is an antioxidant capable to inhibit peroxidative processes protecting cells, DNA and protein from oxidative damage. It also activates TET enzymes allowing the conversion of 5-methylcytosine to 5-hydroximethylcytosine. The water-soluble vitamin C synergistically works with hydrophobic vitamin E further acting as antioxidants [35]. A recent meta-analysis of genome wide methylation studies in response to vitamin C and E availability identified hundreds of genes hypomethylated in the presence of vitamin C and E. Notably, the results were consistent only after stratification of the cohort for age, sex and smoking status thereby confirming those as critical factors [36].

Several other compounds, generally of low molecular weight, found in foods of plant origin have been identified for their biological function possibly through epigenetics reprogramming. These so-called phytochemicals were classified as: polyphenols, phytosterols, terpenoids, alkaloids, and other compounds (including glucosinolates, proteinase inhibitors, etc.).

A role in human health in the prevention of aging and age-related diseases such as diabetes, osteoporosis, cancer and cardiovascular disease has been claimed, likely due to their anti-inflammatory, antioxidant, neuroprotective, chemo-preventive agents. Many review fully covered this topic [37,38] we only mention some of them for their clear role in epigenome regulation.

Spermidine has been identified as an autophagy-inducer in several model organisms and many beneficial effects have been attributed to spermidine supplementation [39]. The molecular mechanism of its action has been linked to inhibition of protein acetyltransferases [40]. Spermidine is produced by the body, but it can also be produced by the intestinal microbiota. Unfortunately, the self-production of spermidine tends to diminish during aging and supplementation with spermidine-containing foods such as wheatgerm, mushrooms, soybeans, aged cheese and others become useful to counteract the incidence of age-related diseases. Inhibition of Histone acetyltransferase (HAT) has been observed after spermidine administration in several model systems. This effect is accompanied with increased resistance to multiple stresses and increased longevity [40]. Recently it was demonstrated in a mice model of amyotrophic lateral sclerosis that spermidine administration restored H3K4me2 levels delaying the onset of the disease and ameliorating the performances of affected mice [41]. Mechanistically it was demonstrated that polyamines treatment increases translation of GCN5 and HAT1 through miR-7648-5p and the 5’-UTR of GCN5 mRNA interaction [41]. 

Many other molecules have been identified for their ability to affect the epigenome, between them the epigallocatechin gallate (EGCG) from the green tea has been one of the best characterized. It is an inhibitor of the DNMT and HDAC. Targets include histones H3 and H4, NF-kB, IL-6, SUZ12/HAT, HDAC, HMT, P16INK4a, RNRβ, RECK1, hTERT, WIF-1, RXRα, RXRβ, CDX2/DNMTI, Bcl-2. Many positive biological effects have been associated with EGCG treatment such as cancer prevention, diabetes prevention and cardiovascular diseases prevention [42,43,44]. 

Broccoli sprouts and cabbages contain the phytochemical phenethyl isothiocyanate, Indole-3-carbinol and diindolylmethane with a role in epigenetics regulation probably affecting HDAC activity [45]. In addition, low doses of sulforaphane associates with human telomerase reverse transcriptase inhibition and DNMTs decreased expression [46]. 

One of the natural best-studied substance affecting the epigenome is Resveratrol (3,5,4’-trihydroxy-trans-stilbene] [47]. It is contained in peanuts, grapes, wine, especially red, and berries. Longer lifespan has been observed in simple model organisms as well as in mammals after its administration. Dietary supplementation associates with decreased level of inflammatory markers and increased insulin sensitivity [48]. The mechanism involves histone modification, DNA methylation and miRNA expression [49] mainly through modulation of SIRT1 activity [49]. In addition, a mechanism involving NF-kB inactivation and FOXO deacetylation have been suggested as a molecular explanation of EGCG role in prostate and breast cancer protection [50]. Other modulators of SIRT1 are known. Examples are quercetin, curcumin and catechins, identified for their anti-inflammatory properties possibly inhibiting COX-2, iNOS and several adhesion molecules likely through NF-kB and AP-1 suppression [51,52]. 

Acetylation of H3 and H4 and DNMT regulation has also been demonstrated with curcumin treatment. A mechanism involving SP1, PTEN but also miRNA has been suggested [53,54]. Genistein, a soy isoflavone, inhibits DNMT1 but has also a role in hTERT, SIRT1, p16, p21, PTEN, p53, and FOXO3A regulation through epigenome reshape [24]. However, follow up studies, including clinical trials, have been disappointing so far [55] (Table 2).

## 3. Dietary Patterns Affecting Epigenome

### 3.1. Overfeeding

Neonatal overfeeding in Wistar rats altered the methylation status of the promoter of the gene coding for pro-opiomelanocortin (POMC) in the hypothalamus. The promoter was more methylated at the binding sites for the transcription factors Sp1 and NF-κB, causing reduced gene expression. 

This result is counterintuitive since the rise of insulin and leptin levels determined by overnutrition are expected to increase POMC expression [56].

The most plausible explanation implies epigenetic programming for the activation of glucocorticoid receptors.

This is supported by the observation that overnutrition in SL kittens increases corticosterone release [57].

It must also be observed that a diet high in sugars and fats induces epigenetic chromatin alterations through cellular metabolism modification, as fats and carbohydrates are converted by mitochondrial energy metabolism and glycolysis into important cofactors such as ATP, acetyl-CoA, SAM, FAD, NAD+ and NADH. These cofactors are used by epigenetic enzymes to regulate processes such as DNA methylation and post-translational modifications of histones [58].

Thus, global and local variations in the concentrations of these cofactors have an effect on the activity of epigenetic enzymes and consequently on the expression of specific genes.

Rathmell et al. proposed a mechanism that is able to explain the involvement of glucose metabolism in histone acetylation. [59].

When glucose meets or exceeds the cell’s energy requirements, it induces an increase in glycolysis, which in turn leads to an increase in citrate synthesis at the mitochondrial level.

The citrate produced in this way can undertake different fates: it can be transported to the cytoplasm, where it is converted into acetyl-CoA by the action of adenosine triphosphate (ATP)-citrate lyase (ACL) and thus serves as a substrate for fatty acid synthesis [60].

Or differently, citrate, being a small molecule, can diffuse into the nucleus through the nuclear pores where, again through the action of ACL, it is converted into acetyl-CoA [26].

In this cellular compartment, acetyl-CoA is used by histone acetyltransferases (HAT) to modify chromatin structure.

In support of this hypothesis, Wellen et al. demonstrated using a small interfering RNA, which silenced ACL, that the amount of global histone acetylation was indeed significantly reduced in several mammalian cell types.

They also showed that the expression of genes involved in the regulation of glucose metabolism was altered [61].

Thus, ACL probably has a key role not only in regulating metabolism but also in regulating epigenetic modifications by providing the necessary substrate for histone acetylation [58,59,60].

### 3.2. Calorie Restriction

Calorie restriction without malnutrition is a dietary treatment where a 20–40% reduction in calorie uptake is not accompanied with nutrient deficiencies. First discovered by McCay in 1935 as capable to increase lifespan up to 40% in rats [62], this treatment increases the lifespan in all model systems tested, from yeast to monkeys [63] and systemic health benefits are also observed in humans [64].

Accumulating evidence in cell cultures and several model systems strongly suggest the ability of calorie restriction without malnutrition to positively impact the detrimental effects of aging process delaying or even avoiding age-associated-diseases like cancer, cardiovascular diseases and diabetes thus resulting in lifespan extension [65]. However, despite the clear metabolic effect and its consistency in every model system from unicellular yeast to monkey, the mechanism by which this remarkable effect is obtained remained somehow elusive [66]. Recent evidence suggests that CR can exerts its effects modulating the mTOR and insulin/IGF1 signaling pathways, DNA methylation and histone deacetylation [67].

Both DNA methylation and post-translational modifications of histones are affected by CR [67].

Older rats treated with CR showed an epigenetic pattern like their younger counterpart suggesting a rejuvenation of the epigenetic pattern during short term calorie restriction, [68]. Alteration of whole blood DNA methylation was observed in obese subjects after calorie restriction and clinical trials have confirmed the efficacy and the involvement of epigenome reshape. Participants underwent weight loss, reduced inflammation and improved cardiometabolic health [68]. Larger studies on non-obese subjects have confirmed the altered epigenome observed in obese subjects. More in depth analysis revealed the involvement of genes responsible for insulin production, inflammation, glucose tolerance, and some signal transduction pathways during calorie restriction treatment [69]. It is interesting to note that in Drosophila melanogaster, offspring of dietary restricted showed increased body weight, resistance to multiple stresses and longer life [70].

A global DNA demethylation accompanied with hypermethylation of some promoter regions has been observed during aging [71,72]. It has been suggested that CR delays aging rescuing DNA methylation pattern associated with aging. An example is the observation that the Ras protooncogene is hypermethylated in response to CR in pancreatic acinar cells with a mechanism based on DNMT [73]. 

In vitro experiments associated glucose restriction, a condition resembling calorie restriction, with hypermethylation of the promoter region of the gene coding for p16INK4a impairing the binding site for E2F-1 [74]. p16INK4a is considered a marker of senescence and aging since its expression increases in tissues overtime [75] and thus the CR-associated downregulation of p16INK4a is a likely cause of the longer lifespan observed. 

On the contrary immortalized lung fibroblasts increases p16INK4a expression when calorie restricted, suggesting that normal and cancer cells respond to calorie restriction differently.

An increase in the activity of histone deacetylases (HDACs) is also observed, suggesting that global deacetylation may play a protective role against metabolic stress and may thus prevent those cellular mechanisms, such as oxidative stress and DNA damage, that underlie aging [76].

Moreover, CR increases intracellular NAD+ concentrations resulting in the activation of a class of NAD+-dependent histone deacetylases (HDACs) known as Sirtuin 1 (SIRT1) [77].

SIRT1 (mammals) and its orthologues in other species, such as SIR2 in yeast, are believed to be key regulators of aging and mediate the action of CR in extending lifespan by increasing resistance to oxidative stress and influencing the DNA repair processes [78]. Recent studies in mice identified around 2000 loci undergoing variation on H3K27ac in response to CR treatment with 850 loss and 1372 gain regions. A combination of metabolomic and subsequent gene ontology analysis pointed to fatty acid and metabolism of carbohydrate as the main target of this epigenetic remodeling and interestingly demonstrated that it was partly dependent on gut microbiota modification upon CR treatment [79].

### 3.3. Ketogenic Diets

It consists of a group of diets with a very low carbohydrate amount, counterbalanced by high fat and normal protein level. The goal of this macronutrient ratio is to trigger the usage of fat instead of glucose as the main cellular fuel. Ketogenic diet (KD) is accompanied with an increase in beta-hydroxybutirate concentration, the major ketone body in animals whose urinary level is used to monitor the metabolic change expected during KD. Historically this dietary approach was used to treat drug-resistant pediatric epilepsy. Interestingly it was also demonstrated that KD has a protein-sparing effect resulting in reduced muscle protein breakdown and ultimately sparing body lean mass during diet. The resulting decrease in insulin level caused by carbohydrate depletion stimulates gluconeogenesis to fuel the brain and eventually increases the usage of liver-produced ketone bodies by all tissues including the brain. In addition, the anorexic properties of ketone bodies reduce the hunger increasing the patient’s compliance. Increasing evidence suggests that metabolic effects observed in response to ketogenic diets are dependent on epigenome reshape. A study on methylome after KD in obese subjects comparing 850,000 CpG identified around 800 genes whose methylation pattern was reverted by KD to that of non-obese subjects. Most of the genes identified are involved in metabolic processes, muscle development and metabolism of proteins [80]. 

### 3.4. Intermittent Fasting

Intermittent fasting encompasses three different dietary treatments, alternate day fasting, 5:2 diet and time restricted feeding. In the first approach a fast day, with zero to severely restricted calorie uptake, is alternated with an ad libitum calorie day. The 5:2 is a modified version of the alternate day fasting consisting of 2 days fasting weekly. Finally, the time restricted feeding suggests concentrating the food uptake within a limited number of hours, typically ranging from 4 to 8. There is a large amount of literature suggesting alternate day fasting ameliorates metabolic performances and induce stable weight loss [81]. Although promoter DNA methylation pattern appears relatively stable, robust oscillation in histone modifications have been associated with circadian rhythm leading to transcriptional waves of selected genes. Binge eating, night shift and eating for prolonged time of the day alter this circadian rhythm while time restricted feeding is capable to restore it, positively affecting health biomarkers and leading to reduced body weight [82]. In addition, a recent study on the metabolic effects of time restricted feeding identified 14 miRNA whose expression level were affected by the treatment. Gene onthology revealed between them the usual culprits like glucose response, autophagy and cellular senescence pathways [83].

### 3.5. Heritage of Dietary-Induced Epigenome

Epidemiological observations, made in several experimental systems as well as in humans, suggest that the environment during fetal development can modulate the risk of developing several diseases such as hypertension, diabetes, obesity and altered lipid profile [84,85,86,87]. The related mechanism points to epigenetic alteration as a possible molecular explanation. One of the first clue of dietary-induced epigenome heritability comes from the observation that rat mothers fed with a low protein diet (9% vs 18%) had fetuses with reduced growth accompanied with DNA hypermethylation in hepatocytes [87]. 

Low protein diet during pregnancy was also associated with hypermethylation of the promoter of the gene coding for the liver oxysterol receptor α in the offspring. This altered methylation pattern was responsible for the reduced expression of this gene in the liver eventually leading to altered cholesterol metabolism. In fact, its altered expression caused reduced expression of Abca1, Abcg5 and Abcg8 genes with impaired efflux of cholesterol and plant sterol into the bile [88]. Methylation sensitive PCR experiments revealed also hypomethylation of glucocorticoid receptor (GR) and peroxisome proliferator-activated receptor α (PPAR α) in the offspring of protein-restricted rat mothers which was interestingly reverted by folic acid supplementation [89] thus enhancing the role of the 1-carbon metabolism in regulating methylation of DNA [90]. The resulting altered expression of PPAR α had several metabolic consequences since the reduced conversion of cortisone to cortisol, due to the PPAR α-dependent inhibition of the 11 β-HSD-1 enzyme, altered carbohydrate, lipid and protein metabolism [91,92,93,94]. This transgenerational transmission of epigenetic pattern resulting from low protein diet was also observed in F2 generation [93,94]. 

Beside low protein diet, high-fat maternal diet during pregnancy altered the methylation pattern of dopamine and opioid-related genes in the offspring [95,96]. 

The resulting altered expression of these genes leads to an increased risk of the onset of obesity and metabolic syndrome. High fat diet during pregnancy leads to increase in fetal fat mass with epigenome modification of the hypothalamus affecting body weight and energy management mainly through leptin receptor modification, neuropeptide y and POMC alteration [96]. 

## 4. Conclusions

Aging and disease susceptibility are highly complex phenotypes. Twin studies suggest a 25% contribution of genetics to longevity thus leaving a 75% to non-heritable determinants [97]. This observation stressed the search for lifestyle interventions capable to positively impact on longevity. Nutrition is certainly a major component as suggested by the relation between single nutrient administration or diet regimens and chronic-degenerative diseases [98,99,100] and by the observation that dietary intervention such as calorie restriction without malnutrition may increase the longevity up to 40% in mammalian model systems [101]. 

The mechanisms by which single nutrient administration or diet regimens may exert this ability is not fully understood but the observation that nutrients, like phytochemicals or diet regimens such as calorie restriction, time restricted feeding or ketogenic diet, may impact on epigenome, regulating thus gene expression, opens to the possibility that nutrition may affect the aging-driven derangement rewinding the epigenetic clock. However great part of the picture is still missing, larger clinical trials are in fact needed to evaluate the efficacy of the different dietary patterns in modulating the epigenome in different populations. Safety and efficacy must also be determined in health and disease and the possibility that specific alleles may regulate the epigenome modulation by nutrients availability ascertained. Additionally, most of the effects of the mentioned phytochemicals were characterized in vitro or in small animals and need to be confirmed in humans. Interestingly. the recent large validation of the epigenetic clock as a measure of biological vs. chronological aging may be a useful marker to further identify which nutrients and diet regimens are capable to positively counteract the aging-driven epigenome derangement. 

## Figures and Tables

**Table 1:** Metabolism–epigenome machinery relationship.

Enzyme:	Function	Cofactor	Substances Affecting Cofactor Availability	Reference
DNMTs	Methylation of DNA	SAM	Met, Folate, Homocysteine Glycolysis, Pentose Pathway Folate, B6, B12, Riboflavin, Betaine, Choline, Glycine	[11,12,13,14,15] [11,21,25]
Demethylases	Demethylation of DNA	a-KG	Krebs cycle, BCAA, Glu, Ser, Asp	[11,21,25]
TET enzymes	Oxidation of methylated DNA	a-KG	Krebs cycle, BCAA, Glu, Ser, Asp	[11,21,25]
HAT enzymes	Histone acetylation	acetyl-CoA	Glucose, acetate	[26]
HDAC enzymes	Histone deacetylation	NAD+	glucose, ketosis, calorie restriction	[27]

**Table 2:** Principal phytochemical with epigenetic targets.

Substance	Target	Reference
Polyamines	miR7648	[39,40,41]
EGCG	DNMT and HDAC	[30,42,44]
Phenethyl		
Isothiocianate	HDAC	[30,45]
Sulforaphane	Telomerase, DNMT	[30,45]
Resveratrol	SIRT1	[30,47,49,50]
Curcumin	DNMT, SIRT1	[30,53,54]
Genistein	DNMT, SIRT1, hTERT	[30]

## Data Availability

Not applicable.

## References

[1] Imamura T., Kerjean A., Heams T., Kupiec J.J., Thenevin C., Pàldi A. (2005). Dynamic CpG and non-CpG methylation of the Peg1/Mest gene in the mouse oocyte and preimplantation embryo. J. Biol. Chem..

[2] Kinde B., Gabel H.W., Gilbert C.S., Griffith E.C., Greenberg M.E. (2015). Reading the unique DNA methylation landscape of the brain: Non-CpG methylation, hydroxymethylation, and MeCP2. Proc. Natl. Acad. Sci. USA.

[3] Patil V., Ward R.L., Hesson L.B. (2014). The evidence for functional non-CpG methylation in mammalian cells. Epigenetics.

[4] Lister R., Pelizzola M., Dowen R.H., Hawkins R.D., Hon G., Tonti-Filippini J., Nery J.R., Lee L., Ye Z., Ngo Q.M. (2009). Human DNA methylomes at base resolution show widespread epigenomic differences. Nature.

[5] Meehan R.R., Thomson J.P., Lentini A., Nestor C.E., Pennings S. (2018). DNA methylation as a genomic marker of exposure to chemical and environmental agents. Curr. Opin. Chem. Biol..

[6] Mehta A., Dobersch S., Romero-Olmedo A.J., Barreto G. (2015). Epigenetics in lung cancer diagnosis and therapy. Cancer Metastasis Rev..

[7] Martin E.M., Fry R.C. (2018). Environmental Influences on the Epigenome: Exposure-Associated DNA Methylation in Human Populations. Annu. Rev. Public Health.

[8] Zhang M., Zhao J., Lv Y., Wang W., Feng C., Zou W., Su L., Jiao J. (2020). Histone Variants and Histone Modifications in Neurogenesis. Trends Cell Biol..

[9] Morgan M.A.J., Shilatifard A. (2020). Reevaluating the roles of histone-modifying enzymes and their associated chromatin modifications in transcriptional regulation. Nat. Genet..

[10] Genchi G., Sinicropi M.S., Lauria G., Carocci A., Catalano A. (2020). The Effects of Cadmium Toxicity. Int. J. Environ. Res. Public Health.

[11] Wang Z., Long H., Chang C., Zhao M., Lu Q. (2018). Crosstalk between metabolism and epigenetic modifications in autoimmune diseases: A comprehensive overview. Cell. Mol. Life Sci..

[12] Ouyang Y., Wu Q., Li J., Sun S., Sun S. (2020). S-adenosylmethionine: A metabolite critical to the regulation of autophagy. Cell Prolif..

[13] Mentch S.J., Locasale J.W. (2016). One-carbon metabolism and epigenetics: Understanding the specificity. Ann. N. Y. Acad. Sci..

[14] Obeid R. (2013). The metabolic burden of methyl donor deficiency with focus on the betaine homocysteine methyltransferase pathway. Nutrients.

[15] Higuchi T., Han Q., Sugisawa N., Yamamoto J., Yamamoto N., Hayashi K., Kimura H., Miwa S., Igarashi K., Bouvet M. (2021). Combination Methionine-methylation-axis Blockade: A Novel Approach to Target the Methionine Addiction of Cancer. Cancer Genom. Proteom..

[16] Rowling M.J., McMullen M.H., Chipman D.C., Schalinske K.L. (2002). Hepatic glycine N-methyltransferase is up-regulated by excess dietary methionine in rats. J. Nutr..

[17] Finkelstein J.D., Martin J.J. (1986). Methionine metabolism in mammals. Adaptation to methionine excess. J. Biol. Chem..

[18] Regina M., Korhonen V.P., Smith T.K., Alakuijala L., Eloranta T.O. (1993). Methionine toxicity in the rat in relation to hepatic accumulation of S-adenosylmethionine: Prevention by dietary stimulation of the hepatic transsulfuration pathway. Arch. Biochem. Biophys..

[19] Waterland R. A. (2006). Assessing the effects of high methionine intake on DNA methylation. J. Nutr..

[20] Mahmoud A.M., Ali M.M. (2019). Methyl Donor Micronutrients that Modify DNA Methylation and Cancer Outcome. Nutrients.

[21] Jiang J., Srivastava S., Liu S., Seim G., Claude R., Zhong M., Cao S., Davé U., Kapur R., Mosley A.L. (2023). Asparagine starvation suppresses histone demethylation through iron depletion. iScience.

[22] Amin S.A., Khatun S., Gayen S., Das S., Jha T. (2023). Are inhibitors of histone deacetylase 8 (HDAC8) effective in hematological cancers especially acute myeloid leukemia (AML) and acute lymphoblastic leukemia (ALL)?. Eur. J. Med. Chem..

[23] Rubio K., Castillo-Negrete R., Barreto G. (2020). Non-coding RNAs and nuclear architecture during epithelial-mesenchymal transition in lung cancer and idiopathic pulmonary fibrosis. Cell. Signal..

[24] Ross S.A. (2003). Diet and DNA methylation interactions in cancer prevention. Ann. N. Y. Acad. Sci..

[25] Filipowicz W., Bhattacharyya S.N., Sonenberg N. (2008). Mechanisms of post-transcriptional regulation by microRNAs: Are the answers in sight?. Nat. Rev. Genet..

[26] Wellen K.E., Hatzivassiliou G., Sachdeva U.M., Bui T.V., Cross J.R., Thompson C.B. (2009). ATP-citrate lyase links cellular metabolism to histone acetylation. Science.

[27] Walker M.A., Tian R. (2018). NAD(H) in mitochondrial energy transduction: Implications for health and disease. Curr. Opin. Physiol..

[28] Chávez-Hidalgo L.P., Martín-Fernández-de-Labastida S., de Pancorbo M.M., Arroyo-Izaga M. (2023). Influence of methyl donor nutrients as epigenetic regulators in colorectal cancer: A systematic review of observational studies. World J. Gastroenterol..

[29] Davis C.D., Uthus E.O. (2004). DNA methylation, cancer susceptibility, and nutrient interactions. Exp. Biol. Med..

[30] Taormina G., Mirisola M.G. (2015). Longevity: Epigenetic and biomolecular aspects. Biomol. Concepts.

[31] Choi S.W., Friso S., Keyes M.K., Mason J.B. (2005). Folate supplementation increases genomic DNA methylation in the liver of elder rats. Br. J. Nutr..

[32] Friso S., Choi S.W., Girelli D., Mason J.B., Dolnikowski G.G., Bagley P.J., Olivieri O., Jacques P.F., Rosenberg I.H., Corrocher R. (2002). A common mutation in the 5,10-methylenetetrahydrofolate reductase gene affects genomic DNA methylation through an interaction with folate status. Proc. Natl. Acad. Sci. USA.

[33] Herdick M., Carlberg C. (2000). Agonist-triggered modulation of the activated and silent state of the vitamin D(3) receptor by interaction with co-repressors and co-activators. J. Mol. Biol..

[34] Seuter S., Neme A., Carlberg C. (2016). Epigenome-wide effects of vitamin D and their impact on the transcriptome of human monocytes involve CTCF. Nucleic Acids Res..

[35] Keshawarz A., Joehanes R., Ma J., Lee G.Y., Costeira R., Tsai P.C., Masachs O.M., Bell J.T., Wilson R., Thorand B. (2023). Dietary and supplemental intake of vitamins C and E is associated with altered DNA methylation in an epigenome-wide association study meta-analysis. Epigenetics.

[36] Sohel M., Aktar S., Biswas P., Amin M.A., Hossain M.A., Ahmed N., Mim M.I.H., Islam F., Mamun A.A. (2023). Exploring the anti-cancer potential of dietary phytochemicals for the patients with breast cancer: A comprehensive review. Cancer Med..

[37] Wang J., Liu Y.M., Hu J., Chen C. (2023). Trained immunity in monocyte/macrophage: Novel mechanism of phytochemicals in the treatment of atherosclerotic cardiovascular disease. Front. Pharmacol..

[38] Eisenberg T., Abdellatif M., Schroeder S., Primessnig U., Stekovic S., Pendl T., Harger A., Schipke J., Zimmermann A., Schmidt A. (2016). Cardioprotection and lifespan extension by the natural polyamine spermidine. Nat. Med..

[39] Eisenberg T., Knauer H., Schauer A., Büttner S., Ruckenstuhl C., Carmona-Gutierrez D., Ring J., Schroeder S., Magnes C., Antonacci L. (2009). Induction of autophagy by spermidine promotes longevity. Nat. Cell Biol..

[40] Sakamoto A., Terui Y., Uemura T., Igarashi K., Kashiwagi K. (2020). Polyamines regulate gene expression by stimulating translation of histone acetyltransferase mRNAs. J. Biol. Chem..

[41] Choi S.H., Yousefian-Jazi A., Hyeon S.J., Nguyen P.T.T., Chu J., Kim S., Kim S., Ryu H.L., Kowall N.W., Ryu H. (2022). Modulation of histone H3K4 dimethylation by spermidine ameliorates motor neuron survival and neuropathology in a mouse model of ALS. J. Biomed. Sci..

[42] Brimson J.M., Prasanth M.I., Kumaree K.K., Thitilertdecha P., Malar D.S., Tencomnao T., Prasansuklab A. (2022). Tea Plant (*Camellia sinensis*): A Current Update on Use in Diabetes, Obesity, and Cardiovascular Disease. Nutrients.

[43] Wong S.C., Kamarudin M.N.A., Naidu R. (2023). Anticancer Mechanism of Flavonoids on High-Grade Adult-Type Diffuse Gliomas. Nutrients.

[44] Wang L., Li P., Feng K. (2023). EGCG adjuvant chemotherapy: Current status and future perspectives. Eur. J. Med. Chem..

[45] Li Y., Buckhaults P., Li S., Tollefsbol T. (2018). Temporal Efficacy of a Sulforaphane-Based Broccoli Sprout Diet in Prevention of Breast Cancer through Modulation of Epigenetic Mechanisms. Cancer Prev. Res..

[46] Vrânceanu M., Galimberti D., Banc R., Dragoş O., Cozma-Petruţ A., Hegheş S.C., Voştinaru O., Cuciureanu M., Stroia C.M., Miere D. (2022). The Anticancer Potential of Plant-Derived Nutraceuticals via the Modulation of Gene Expression. Plants.

[47] Bertelli A.A., Das D.K. (2009). Grapes, wines, resveratrol, and heart health. J. Cardiovasc. Pharmacol..

[48] Hernández-Lepe M.A., Ortiz-Ortiz M., Hernández-Ontiveros D.A., Mejía-Rangel M.J. (2023). Inflammatory Profile of Older Adults in Response to Physical Activity and Diet Supplementation: A Systematic Review. Int. J. Environ. Res. Public Health.

[49] Chung S., Yao H., Caito S., Hwang J.W., Arunachalam G., Rahman I. (2010). Regulation of SIRT1 in cellular functions: Role of polyphenols. Arch. Biochem. Biophys..

[50] Chen Q., Ganapathy S., Singh K.P., Shankar S., Srivastava R.K. (2010). Resveratrol induces growth arrest and apoptosis through activation of FOXO transcription factors in prostate cancer cells. PLoS ONE.

[51] Zhang R., Chen H.Z., Liu J.J., Jia Y.Y., Zhang Z.Q., Yang R.F., Zhang Y., Xu J., Wei Y.S., Liu D.P. (2010). SIRT1 suppresses activator protein-1 transcriptional activity and cyclooxygenase-2 expression in macrophages. J. Biol. Chem..

[52] Biesalski H.K. (2007). Polyphenols and inflammation: Basic interactions. Curr. Opin. Clin. Nutr. Metab. Care.

[53] Ghorbaninejad M., Khademi-Shirvan M., Hosseini S., Meyfour A., Shahhoseini M., Baghaban Eslaminejad M. (2023). Effective role of Curcumin on expression regulation of EZH2 histone methyltransferase as a dynamic epigenetic factor in osteogenic differentiation of human mesenchymal stem cells. Biochim. Biophys. Acta Gene Regul. Mech..

[54] Ming T., Tao Q., Tang S., Zhao H., Yang H., Liu M., Ren S., Xu H. (2022). Curcumin: An epigenetic regulator and its application in cancer. Biomed. Pharmacother..

[55] Yang C.S., Chen J.X., Wang H., Lim J. (2016). Lessons learned from cancer prevention studies with nutrients and non-nutritive dietary constituents. Mol. Nutr. Food Res..

[56] Rajaselvi N.D., Jida M.D., Ajeeshkumar K.K., Nair S.N., John P., Aziz Z., Nisha A.R. (2023). Antineoplastic activity of plant-derived compounds mediated through inhibition of histone deacetylase: A review. Amino Acids.

[57] McGowan P.O., Sasaki A., D’Alessio A.C., Dymov S., Labonté B., Szyf M., Turecki G., Meaney M.J. (2009). Epigenetic regulation of the glucocorticoid receptor in human brain associates with childhood abuse. Nat. Neurosci..

[58] Taormina G., Russo A., Latteri M.A., Mirisola M.G. (2019). Mitochondrion at the Crossroad Between Nutrients and Epigenome. Front. Endocrinol..

[59] Rathmell J.C., Newgard C.B. (2009). Biochemistry. A glucose-to-gene link. Science.

[60] Hatzivassiliou G., Zhao F., Bauer D.E., Andreadis C., Shaw A.N., Dhanak D., Hingorani S.R., Tuveson D.A., Thompson C.B. (2005). ATP citrate lyase inhibition can suppress tumor cell growth. Cancer Cell.

[61] Icard P., Wu Z., Fournel L., Coquerel A., Lincet H., Alifano M. (2020). ATP citrate lyase: A central metabolic enzyme in cancer. Cancer Lett..

[62] McCay C.M., Crowell M.F., Maynard L.A. (1989). The effect of retarded growth upon the length of life span and upon the ultimate body size. Nutrition.

[63] Taormina G., Mirisola M.G. (2014). Calorie restriction in mammals and simple model organisms. BioMed Res. Int..

[64] Most J., Tosti V., Redman L.M., Fontana L. (2017). Calorie restriction in humans: An update. Ageing Res. Rev..

[65] Ribarič S. (2012). Diet and aging. Oxidative Med. Cell. Longev..

[66] Lee C.K., Klopp R.G., Weindruch R., Prolla T.A. (1999). Gene expression profile of aging and its retardation by caloric restriction. Science.

[67] Kim C.H., Lee E.K., Choi Y.J., An H.J., Jeong H.O., Park D., Kim B.C., Yu B.P., Bhak J., Chung H.Y. (2016). Short-term calorie restriction ameliorates genomewide, age-related alterations in DNA methylation. Aging Cell.

[68] Belsky D.W., Huffman K.M., Pieper C.F., Shalev I., Kraus W.E. (2017). Change in the Rate of Biological Aging in Response to Caloric Restriction: CALERIE Biobank Analysis. J. Gerontol. Ser. A Biol. Sci. Med. Sci..

[69] Ramaker M.E., Corcoran D.L., Apsley A.T., Kobor M.S., Kraus V.B., Kraus W.E., Lin D.T.S., Orenduff M.C., Pieper C.F., Waziry R. (2022). Epigenome-wide Association Study Analysis of Calorie Restriction in Humans, CALERIETM Trial Analysis. J. Gerontol. Ser. A Biol. Sci. Med. Sci..

[70] Lee H.Y., Lee B., Lee E.J., Min K.J. (2023). Effects of Parental Dietary Restriction on Offspring Fitness in *Drosophila melanogaster*. Nutrients.

[71] Wilson V.L., Jones P.A. (1983). DNA methylation decreases in aging but not in immortal cells. Science.

[72] Waki T., Tamura G., Sato M., Motoyama T. (2003). Age-related methylation of tumor suppressor and tumor-related genes: An analysis of autopsy samples. Oncogene.

[73] Hass B.S., Hart R.W., Lu M.H., Lyn-Cook B.D. (1993). Effects of caloric restriction in animals on cellular function, oncogene expression, and DNA methylation in vitro. Mutat. Res..

[74] Li Y., Liu L., Tollefsbol T.O. (2010). Glucose restriction can extend normal cell lifespan and impair precancerous cell growth through epigenetic control of hTERT and p16 expression. FASEB J. Off. Publ. Fed. Am. Soc. Exp. Biol..

[75] Krishnamurthy J., Torrice C., Ramsey M.R., Kovalev G.I., Al-Regaiey K., Su L., Sharpless N.E. (2004). Ink4a/Arf expression is a biomarker of aging. J. Clin. Investig..

[76] Leibiger I.B., Berggren P.O. (2006). Sirt1: A metabolic master switch that modulates lifespan. Nat. Med..

[77] Cohen H.Y., Miller C., Bitterman K.J., Wall N.R., Hekking B., Kessler B., Howitz K.T., Gorospe M., de Cabo R., Sinclair D.A. (2004). Calorie restriction promotes mammalian cell survival by inducing the SIRT1 deacetylase. Science.

[78] Lin S.J., Defossez P.A., Guarente L. (2000). Requirement of NAD and SIR2 for life-span extension by calorie restriction in Saccharomyces cerevisiae. Science.

[79] Fan Y., Qian H., Zhang M., Tao C., Li Z., Yan W., Huang Y., Zhang Y., Xu Q., Wang X. (2023). Caloric restriction remodels the hepatic chromatin landscape and bile acid metabolism by modulating the gut microbiota. Genome Biol..

[80] Crujeiras A.B., Izquierdo A.G., Primo D., Milagro F.I., Sajoux I., Jácome A., Fernandez-Quintela A., Portillo M.P., Martínez J.A., Martinez-Olmos M.A. (2021). Epigenetic landscape in blood leukocytes following ketosis and weight loss induced by a very low calorie ketogenic diet (VLCKD) in patients with obesity. Clin. Nutr..

[81] Trepanowski J.F., Kroeger C.M., Barnosky A., Klempel M.C., Bhutani S., Hoddy K.K., Gabel K., Freels S., Rigdon J., Rood J. (2017). Effect of Alternate-Day Fasting on Weight Loss, Weight Maintenance, and Cardioprotection Among Metabolically Healthy Obese Adults: A Randomized Clinical Trial. JAMA Intern. Med..

[82] Vollmers C., Schmitz R.J., Nathanson J., Yeo G., Ecker J.R., Panda S. (2012). Circadian oscillations of protein-coding and regulatory RNAs in a highly dynamic mammalian liver epigenome. Cell Metab..

[83] Saini S.K., Singh A., Saini M., Gonzalez-Freire M., Leeuwenburgh C., Anton S.D. (2022). Time-Restricted Eating Regimen Differentially Affects Circulatory miRNA Expression in Older Overweight Adults. Nutrients.

[84] Langley S.C., Jackson A.A. (1994). Increased systolic blood pressure in adult rats induced by fetal exposure to maternal low protein diets. Clin. Sci..

[85] Dahri S., Snoeck A., Reusens-Billen B., Remacle C., Hoet J.J. (1991). Islet function in offspring of mothers on low-protein diet during gestation. Diabetes.

[86] Ravelli G.P., Stein Z.A., Susser M.W. (1976). Obesity in young men after famine exposure in utero and early infancy. N. Engl. J. Med..

[87] Roseboom T.J., van der Meulen J.H., Ravelli A.C., Osmond C., Barker D.J., Bleker O.P. (2001). Effects of prenatal exposure to the Dutch famine on adult disease in later life: An overview. Mol. Cell. Endocrinol..

[88] Rees W.D., Hay S.M., Brown D.S., Antipatis C., Palmer R.M. (2000). Maternal protein deficiency causes hypermethylation of DNA in the livers of rat fetuses. J. Nutr..

[89] Van Straten E.M., Bloks V.W., Huijkman N.C., Baller J.F., van Meer H., Lütjohann D., Kuipers F., Plösch T. (2010). The liver X-receptor gene promoter is hypermethylated in a mouse model of prenatal protein restriction. Am. J. Physiol. Regul. Integr. Comp. Physiol..

[90] Lillycrop K.A., Phillips E.S., Jackson A.A., Hanson M.A., Burdge G.C. (2005). Dietary protein restriction of pregnant rats induces and folic acid supplementation prevents epigenetic modification of hepatic gene expression in the offspring. J. Nutr..

[91] He A., Chen X., Tan M., Chen Y., Lu D., Zhang X., Dean J.M., Razani B., Lodhi I.J. (2020). Acetyl-CoA Derived from Hepatic Peroxisomal β-Oxidation Inhibits Autophagy and Promotes Steatosis via mTORC1 Activation. Mol. Cell.

[92] Tugwood J.D., Issemann I., Anderson R.G., Bundell K.R., McPheat W.L., Green S. (1992). The mouse peroxisome proliferator activated receptor recognizes a response element in the 5′ flanking sequence of the rat acyl CoA oxidase gene. EMBO J..

[93] Hermanowski-Vosatka A., Gerhold D., Mundt S.S., Loving V.A., Lu M., Chen Y., Elbrecht A., Wu M., Doebber T., Kelly L. (2000). PPARalpha agonists reduce 11beta-hydroxysteroid dehydrogenase type 1 in the liver. Biochem. Biophys. Res. Commun..

[94] Burdge G.C., Slater-Jefferies J., Torrens C., Phillips E.S., Hanson M.A., Lillycrop K.A. (2007). Dietary protein restriction of pregnant rats in the F0 generation induces altered methylation of hepatic gene promoters in the adult male offspring in the F1 and F2 generations. Br. J. Nutr..

[95] Vucetic Z., Kimmel J., Totoki K., Hollenbeck E., Reyes T.M. (2010). Maternal high-fat diet alters methylation and gene expression of dopamine and opioid-related genes. Endocrinology.

[96] Harmancıoğlu B., Kabaran S. (2023). Maternal high fat diets: Impacts on offspring obesity and epigenetic hypothalamic programming. Front. Genet..

[97] Brooks-Wilson A.R. (2013). Genetics of healthy aging and longevity. Hum. Genet..

[98] Kumar A., Chinnathambi S., Kumar M., Pandian G.N. (2023). Food Intake and Colorectal Cancer. Nutr. Cancer.

[99] Peña-Jorquera H., Cid-Jofré V., Landaeta-Díaz L., Petermann-Rocha F., Martorell M., Zbinden-Foncea H., Ferrari G., Jorquera-Aguilera C., Cristi-Montero C. (2023). Plant-Based Nutrition: Exploring Health Benefits for Atherosclerosis, Chronic Diseases, and Metabolic Syndrome—A Comprehensive Review. Nutrients.

[100] Suzuki M., Wilcox B.J., Wilcox C.D. (2001). Implications from and for food cultures for cardiovascular disease: Longevity. Asia Pac. J. Clin. Nutr..

[101] Fontana L. (2018). Interventions to promote cardiometabolic health and slow cardiovascular ageing. Nat. Rev. Cardiol..

